# The Impact of Auricular Vagus Nerve Stimulation on Pain and Life Quality in Patients with Fibromyalgia Syndrome

**DOI:** 10.1155/2020/8656218

**Published:** 2020-02-28

**Authors:** Nazlı Kutlu, Ali Veysel Özden, Hasan Kerem Alptekin, Jülide Öncü Alptekin

**Affiliations:** ^1^Beykoz State Hospital, Physiotherapy and Rehabilitation Department, Turkey; ^2^Bahçeşehir University, Health Sciences Institute, Turkey; ^3^Şişli Etfal Education and Research Hospital, Physical Medicine and Rehabilitation Department, Turkey

## Abstract

The purpose of this study is to evaluate the impact of auricular vagus nerve stimulation, applied in conjunction with an exercise treatment program, on pain and life quality in patients with fibromyalgia syndrome (FMS). To achieve the study objectives, 60 female patients between the ages 18 and 50, with diagnosed FMS according to the American College of Rheumatology (ACR) 2010 diagnostic criteria, were randomly divided into 2 groups of 30. The first group was assigned 20 sessions of a home-based exercise program, while the second group was assigned 20 sessions of auricular vagus nerve stimulation and 20 sessions of a home-based exercise program. Patients were assessed before and after the treatments using the Visual Analog Scale (VAS) for pain, Beck Depression Scale for depression, Beck Anxiety Scale for anxiety, Fibromyalgia Impact Questionnaire (FIQ) for functional evaluation, and Short Form-36 (SF-36) for life quality. In this randomized controlled trial, comparisons within the groups revealed that both groups had statistically significant improvements in pain, depression, anxiety, functionality, and life quality scores (*p* < 0.05), while comparisons across the groups revealed that the group experiencing the vagus nerve stimulation had no statistically significant differences between the baseline scores, except for those of SF-36's subparameters of physical function, social functionality, and pain. In fact, comparisons across the groups after the interventions revealed that the group experiencing the vagus nerve stimulation had better scores but not statistically significant. From analysis of this data, we observed that vagus nerve stimulation in FMS treatment did not give additional benefit together with exercise, except for three subparameters of SF-36. It was identified that further studies which separately investigate the effects of vagus nerve stimulation and exercise on FMS with longer follow-up periods and an increased number of patients are needed.

## 1. Introduction

Fibromyalgia syndrome (FMS) is a syndrome characterized by chronic widespread pain that continues for a minimum of 3 months and pain felt upon 11 out of 9 pairs of identified sore points. It is often accompanied by systemic symptoms such as fatigue, sleep disorders, cognitive dysfunction, and depression [1, 2]. Moreover, headache, dysmenorrhea, irritable bowel syndrome, restless leg syndrome, chest pain, jaw pain, stomach ache, sensitive skin, mitral valve prolapses, sicca symptoms, Raynaud phenomenon, and female urethral syndrome accompany the clinical picture in many patients with FMS [2, 3]. This syndrome, the etiology of which is unknown, is commonly seen in women, and the age of onset is usually 30–50 [4]. Its prevalence among the general population is reported to be between 2.9 and 4.7% percent [5]. While it is often seen in the adult population, it can also be observed in childhood or later age [6]. Recent research studies show that various anomalies in genetic structure, autonomic nervous system (ANS), neurotransmitters, hypothalamic-hypophyseal-adrenal axe hormones, oxidative stress, pain modulation centers, central sensitization, and muscular structure occur in FMS [7]. The etiology of FMS is still not fully understood; nonetheless, the data reveals that this syndrome may stem from dysfunctionality in ANS [8]. Sympathetic nervous system (SNS) dominance is widespread in FMS, chronic fatigue, irritable bowel syndrome, and interstitial cystitis [9]. A highly peripheral sympathetic tone causes regional ischemia, which in turn causes widespread pain. Therapeutic interventions that result in vasodilatation (e.g., exercise) and appropriate autonomic changes are proven to be effective for reducing pain levels [10].

The “great and perfect protector” of the body, the vagus nerve, includes a neuro-endocrine-immune network that ensures homeostasis. The vagus nerve, which has reciprocal neural connections with more than one region of the brain, serves a control center that combines sensitive information and provides appropriate feedback responses. Recent studies show that the vagus nerve also encompasses inflammation, mood, and pain regulation. All of these can be modulated with vagus nerve stimulation. Vagus nerve stimulation can generate a neuromodulating effect to activate other natural protective paths to improve health [11]. The development of vagus nerve stimulation began in the 19^th^ century. A number of new electrical stimulation devices have now been developed. Noninvasive transcutaneal devices stimulate the vagus nerve through the auricular branch or from the carotid. Moreover, they are used in the treatment of various disorders, such as epilepsy, pain, and headache [12]. Recent preclinical studies have shown that vagus nerve stimulation heavily modulates pain in humans and is quite effective. The use of a medical device allows the auricular branch of the vagus nerve to be stimulated without surgical intervention. Consequently, it has been found that the threshold of pain is elevated and mechanical pain sensitivity is reduced [13].

As a result of these completed studies, there is a possibility that FMS is a disease, which occurs through ANS impairment. Vagus nerve stimulation could be used as an additional treatment method to improve the ANS impairment. However, as there are no studies that investigate vagus nerve stimulation in FMS, this study is designed and executed.

## 2. Material and Method

Our study, in which we investigated the impact of auricular vagus nerve stimulation on pain and life quality in patients with FMS, was conducted in Beykoz Public Hospital's Department of Physiotherapy and Rehabilitation. A total of 60 female patients within the age range 18–50, with diagnosed FMS by a physiatrist according to 2010 ACR criteria, were included in this study. We include only females to make groups homogenous and ended the age range at 50 to avoid comorbid illnesses seen in elderly people and also menopause. Pregnant, perimenopausal, and postmenopausal women are not included; also those experiencing any comorbid illnesses like neurological deficits, diabetes, neuropathic disorders, chronic inflammation, immune deficiency, cardiac disorders, and current vitamin D intake were excluded from the study. In addition, those who started using new drugs in the last month and during the study were not included. Patients who experienced vasovagal syncope in their past could not participate in the study.

Ethics committee approval was awarded by the Ethics Committee of Beykoz State Hospital Clinical Research prior to the commencement of the study. Written consent forms were obtained from all patients to demonstrate their consent to being included in the study. Patients were assessed twice during the study, first before treatment and then after treatment. Before the assessment, data was obtained from patients relating to age, height, weight, occupation, level of education, marital status, regular medication, general health complaints, and the duration of these complaints. All responses were recorded. Patients were also asked to not participate in any other treatments during the study, in order to prevent any external influences on the data and study parameters.

Patients are diagnosed with FSM, in accordance with the ACR 2010 Diagnostic Criteria. A total of 60 patients are included in the study. Patients were randomly divided into two groups: an exercise (control) group and an exercise and vagus stimulation (treatment) group. Randomization was carried out by random number assignment. The exercise group began with 30 patients, and the exercise and vagus stimulation group began with 30 patients. By the end of the study, there were 25 patients in the exercise group and 27 patients in the exercise and vagus stimulation group. Five patients in the exercise group were excluded as they had not completed the exercise program. Three patients in the exercise and vagus stimulation group failed to complete the study, and they missed treatment sessions. There was no blinding in the study.

The exercise group was assigned a program, which consisted of strengthening, stretching, isometric, and posture exercises, targeting the body and upper and lower extremities. That program was home-based, and the program was requested to be completed. Patients were asked to attend weekly face-to-face sessions with a total of 4 of these sessions in the study duration.

**Figure 1:**
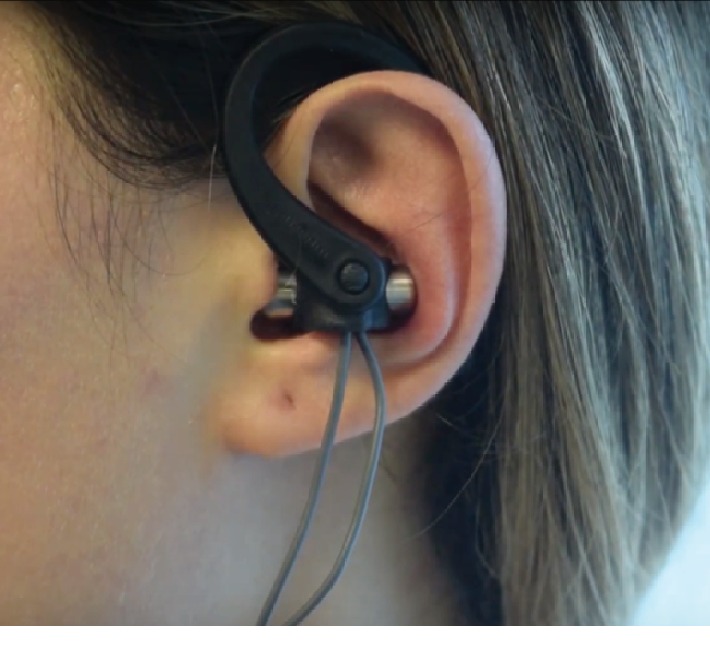
Electrode placing of the auricular vagus nerve stimulation.

Patients in the exercise and vagus stimulation group received auricular vagus nerve stimulation at Beykoz Public Hospital's Department of Physical Therapy and Rehabilitation on 5 weekdays for 4 weeks, making up a total of 20 sessions with each session taking 30 minutes. Patients were admitted to the treatment as day visitors. Vagus nerve stimulation is carried out with a TENS device, which has specially designed surface electrodes in the shape of earphones, the size of which can be selected according to ear size. Electrodes were placed to correspond with the inner and rear surfaces of the tragus and the concha for both ears (Figure 1). The application is carried out, for 30 minutes, using a biphasic, asymmetrical waveform with a pulse duration that is less than 500 microseconds and a frequency of 10 hertz. Amplitude is adjusted according to the sensory threshold level. Patients in this group were also assigned the same home-based exercise program assigned to the patients in the exercise group with application of the program 5 days a week with 2 sets per day, and each set involved 10 repetitions of every exercise. Patients were asked to attend weekly face-to-face sessions with a total of 4 of these sessions in the study duration.

Measurements were performed twice, before and after the treatments. Patients were asked to complete the Turkish version of the Fibromyalgia Impact Questionnaire (FIQ), consisting of 21 questions, displaying the extent to which pain impacts the daily activities, social lives, moods, and professional lives of the patients during the previous week. This scale was developed by Burchardt et al. to gauge the functional condition of patients with FM, while its specific validity and reliability adaptation for Turkey was carried out by Sarmer et al. [14].

Pain intensity was evaluated within the context of this study using the Visual Analog Scale (VAS). Patients were asked to select the corresponding interval for their own intensity of pain on a table of 0-100 mm with the explanation that 0 refers to no pain and 100 is excruciating pain [15].

Life quality of patients was evaluated using the SF-36 life quality scale. The Turkish validity and reliability of this scale was carried out by Koçyiğit et al. Investigating life quality regarding overall health, this questionnaire contains 36 questions in 8 different categories [16, 17].

The level of depression of patients was evaluated using the Beck Depression Scale. The Turkish validity and reliability of this scale was carried out by Hisli in 1988. It is a questionnaire that consists of 21 questions with 4 items, and each item is awarded between 0 and 3 points [18].

The level of anxiety of patients was evaluated using the Beck Anxiety Scale. This scale was developed by Beck et al., and its Turkish validity and reliability studies were conducted by Ulusoy et al. in 1998 [19].

### 2.1. Statistical Analysis

The presentation of variables acquired within the scope of the study such as sex, level of education, and occupation includes numbers (*n*) and percentage values.

The Shapiro-Wilk test is used to determine whether the variables in the study are congruent with the normal distribution. Interquartile range (IQR) values for the variables without normal distribution and average ± SD (Standard Deviation) values for the variables with normal distribution are provided in the descriptive statistics.

In the comparison of measurement values before and after the treatment, results of *t*-test for the variables with normal distribution and of Wilcoxon-Signed Rank test for the variables without normal distribution are provided.

In the comparison of the treatment group and control group after the treatment, results of *t*-test for variables with normal distribution and of Mann–Whitney *U* test for variables without normal distribution are provided.

Chi-square and likelihood ratio chi-square tests were used to assess the relationship between patient and control group complaints, accompanying diseases, duration of pain, and daily sports activities.

IBM SPSS Statistics 21.0 (IBM Corp. Released 2012. IBM SPSS Statistics for Windows, Version 21.0. Armonk, NY: IBM Corp.) and MS-Excel 2007 were used for statistical analyses and calculations. The level for statistical significance was accepted to be *p* < 0.05.

## 3. Findings

The average age of the individuals in the control group is 38.60 ± 9.34 years, while the individuals in the treatment group have an average age of 39.44 ± 8.28 years, and all the respondents are female (*n* = 52). There is no statistically significant difference between the average ages of the individuals in the control group and the treatment group (*t* = 0.346, *p* = 0.731). The individuals in the control group have an average height of 1.64 ± 0.07 meters, while those in the treatment group have 1.60 ± 0.06 meters. A statistically significant difference between the average height of the control group and that of the treatment group is found (*t* = 2,035, *p* = 0.047). The average weight of those in the control group is 69.32 ± 13.86 kg and those in the treatment group have an average weight of 66.78 ± 12.67 kg, and no statistically significant differences were found between groups with respect to weight (*t* = 0.691, *p* = 0.493).

Within the control group, 84.0% (*n* = 21) of the individuals are married and 16.0% (*n* = 14) are single. Among them, 48.0% are housewives and 24.0% are teachers. 72.0% of the respondents complained from pain only. The remaining 28.0%, however, expressed complaints of numbness, weakness, and fatigue, alongside pain. 80.0% of respondents stated that they do not undertake any daily sporting activities, whereas 20.0% stated they take daily walks (Table 1).

Within the individuals in the treatment group, 66.7% (*n* = 18) are married and 22.2% (*n* = 6) are single (Table 2). In this group, 51.9% are housewives and 14.8% are nurses. Pain-only complaints were reported from 66.7% of the patients. The remaining 33.7%, however, expressed complaints such as numbness, weakness, and fatigue, in addition to pain. Similarly, 59.3% of patients stated that they undertake no daily sporting activities and 22.2% reported walking.

The average score from the Beck Depression Scale before the exercise is 16.76 ± 10.63, while the average score after the exercise is detected to be 11.92 ± 7.06 in the control group (Table 3). A statistically significant difference between the average scores from the Beck Depression Scale before and after the exercise is identified (*t* = 4.132, *p* < 0.001). Similarly, the median score from the Beck Anxiety Scale before the exercise was 20.00 (IQR = 16.5) while the median score after the exercise was 13.00 (IQR = 11.00). There is a statistically significant difference between the median scores from the Beck Anxiety Scale before and after the treatment (*Z* = 3.636, *p* < 0.001).

The average score acquired from the Fibromyalgia Impact Questionnaire before the exercise is 54.48 ± 18.81 and the average score from the same questionnaire after the exercise is 41.93 ± 18.15. A statistically significant difference between the scores from the Fibromyalgia Impact Questionnaire before and after the exercise is determined (*t* = 5.763, *p* < 0.001). The average score of the VAS Pain Scale before the exercise is 5.67 ± 2.10, while the average score after the exercise is 3.45 ± 1.73. There is a statistically significant difference between the average scores from the VAS Pain Scale, collected before and after the exercise (*t* = 7.097, *p* < 0.001).

An examination of the scores from the SF-36 Scale reveals that the median score before the exercise under the Physical Function subscale was 70.00 (IQR = 17.50) and the median score after the exercise was 85.00 (IQR = 22.50). A statistically significant difference is identified between the median scores, acquired before and after the exercise (*Z* = 3.619, *p* < 0.001). In the Physical Role Difficulty subscale, the median score before the exercise was 25.00 (IQR = 50.00) and median score after the exercise was 50.00 (IQR = 50.00). A statistically significant difference was identified between the scores, acquired before and after the exercise (*Z* = 3.225, *p* < 0.001). In the Emotional Role Difficulty subscale, the median score before the exercise was 33.3 (IQR = 66.67) and the median score after the exercise was 66.67 (IQR = 33.33). A statistically significant difference was identified between the scores, acquired before and after the exercise (*Z* = 2.336, *p* = 0.019). In the Energy/Liveliness/Vitality subscale, the median score before the exercise was 40.00 (IQR = 37.50) and the median score after the exercise was 50.00 (IQR = 20.00). A statistically significant difference was identified between the scores, acquired before and after the exercise (*Z* = 3.791, *p* < 0.001). In the Mental Health subscale, the average score before the exercise was 50.24 ± 19.26 and the average score after the exercise was 62.40 ± 16.93. There is a statistically significant difference between average scores: scores before the exercise and scores after the exercise (*t* = 3.919, *p* = 0.001). In the Social Functionality subscale, the median score before the exercise was 62.50 (IQR = 25.00) and the median score after the exercise was 75.00 (IQR = 18.75). A statistically significant difference was identified between the medians scores, acquired before and after the exercise (*Z* = 3.824, *p* < 0.001). In the General Health Perception subscale, the average score before the exercise was 42.60 ± 21.51 and the average score after the exercise was 52.20 ± 15.46. There is a statistically significant difference between the average scores, before the exercise and after the exercise (*t* = 4.129, *p* < 0.001). In the Pain subscale, the average score before the exercise was 41.10 ± 21.06 and the average score after the exercise was 59.50 ± 15.28. A statistically significant difference between the average scores before and after the exercise is also determined (*t* = 5.451, *p* < 0.001).

The median of the scores collected using the Beck Depression Scale before the treatment was 16.00 (IQR = 12.00), while the median of the scores from after the treatment was 8.00 (IQR = 12.00). A statistically significant difference was identified between medians of scores, collected using the Beck Depression Scale before and after the treatment (*Z* = 3.660, *p* < 0.001) (Table 4). Similarly, the median score before the treatment collected using the Beck Anxiety Scale was 18.00 (IQR = 13.00) while the median score after the treatment was 13.00 (IQR = 13.00). A statistically significant difference between median scores before and after the treatment as per Beck Anxiety Scale is also identified (*Z* = 3.692, *p* < 0.001).

The average of the before treatment scores, obtained using the FIQ, was 61.98 ± 18.45, and the average of the after treatment score was 37.27 ± 19.48. A statistically significant difference between the average scores acquired using the FIQ before and after treatment was determined (*t* = 5.883, *p* < 0.001). The average before treatment scores, collected using the VAS Pain Scale, was 6.17 ± 2.58, and the average after treatment score was 2.56 ± 1.91. There is a statistically significant difference between the before and after treatment score averages as per VAS Pain Scale (*t* = 5.859, *p* < 0.001).

An examination of the scores from the SF 36 Scale shows that in the Physical Function subscale, median of the before treatment scores was 65.00 (IQR = 25.00) and median of the after treatment scores was 80.00 (IQR = 25.00). There is a statistically significant difference between the median scores, acquired before and after treatment (*Z* = 4.024, *p* < 0.001). In the Physical Role Difficulty, the median of the before treatment scores was 00.00 (IQR = 50.00) and the median of the after treatment scores was 75.00 (IQR = 75.00). There is a statistically significant difference between the medians scores, acquired before and after treatment (*Z* = 3.116, *p* = 0.002). In the Emotional Role Difficulty subscale, the median of the before treatment scores was 0.00 (IQR = 66.67) and the median of the after treatment scores was 100.00 (IQR = 66.67). There is a statistically significant difference between the medians scores, acquired before and after treatment (*Z* = 2.764, *p* = 0.006). In the Energy/Liveliness/Vitality subscale, the average of the before treatment scores was 28.70 ± 21.64 and the average of the after treatment score was 57.59 ± 22.97. A statistically significant difference between the score averages from before and after treatment is identified (*Z* = 5.153, *p* < 0.001). In the Mental Health subscale, the average of the before treatment scores was 46.37 ± 19.09 and the average of the after treatment scores was 65.33 ± 20.66. A statistically significant difference between the score averages from before and after treatment is identified (*t* = 4.265, *p* = 0.001). In the Social Functionality subscale, the average of the before treatment scores was 47.22 ± 26.02 and the average of the after treatment scores was 69.91 ± 22.80. A statistically significant difference between the score averages from before and after treatment is identified (*t* = 3.583, *p* = 0.001). In the General Health Perception subscale, the average of the before treatment scores was 33.89 ± 19.38 and the average of the after treatment score was 56.85 ± 21.45. A statistically significant difference between the score averages from before and after treatment is identified (*t* = 6.126, *p* < 0.001). In the Pain subscale, the average of the before treatment scores was 27.87 ± 21.54 and the average of the after treatment score was 58.05 ± 18.80. A statistically significant difference between the score averages from before and after treatment is identified (*t* = 6.741, *p* < 0.001).

In reviewing the scores, acquired using the SF 36 Scale, a significant difference between the scores of the treatment group and the control group occurs, with respect to the Physical Function subscale (*Z* = 2.281, *p* = 0.023). The median of the scores of the control group from the Physical Function subscale was 70.00 (IQR = 17.50), and the median of the scores of the treatment group was 65.00 (IQR = 25.00). There was no statistically significant difference between the medians of the scores from subscales of Physical Role Difficulty, Emotional Role Difficulty, and Energy/Liveliness/Vitality (respectively: *Z* = 0.908, *p* = 0.364; *Z* = 0.520, *p* = 0.603; and *Z* = 1.444, *p* = 0.149). Similarly, the treatment group and the control group did not reveal any statistically significant differences with respect to their average scores from the Mental Health and General Health Perception subscales (respectively: *t* = 0.727, *p* = 0.471; *Z* = 0.536, *p* = 0.131). Finally, a statistically significant difference between the average of scores from the subscales of Social Functionality and Pain is determined (respectively: *t* = 2.386, *p* = 0.021; *t* = 2.363, *p* = 0.0.18) (Table 5).

In reviewing the scores after the intervention, the treatment group and the control group did not reveal any statistically significant differences (Table 6).

## 4. Discussion and Conclusion

Multiple controlled studies, investigating both pharmacological and nonpharmacological FMS treatments, have been carried out during the past 40 years [20]. Studies found out that the treatment protocols involve methods that approach FMS as a systemic disorder, rather than a regional or multifocal skeletal disorder. Approaches that combine pharmacological and nonpharmacological treatment methods, considering the severity of symptoms, variety, and functional conditions of the patient, are required in order to gain maximum benefit from the treatment [21]. In our study, we aimed to see exercise and vagus nerve stimulation effects on FMS.

A new method, associated with FMS treatment, is the noninvasive stimulation of the vagus nerve, for which research is still ongoing. While the impact mechanism of the vagus nerve stimulation is not yet fully understood, studies on humans reveal that it affected many regions of the brain subcortical and cortical levels [22]. However, there are many unknowns about this procedure.

The best location and parameters of stimulation for the auricular vagus nerve have not been revealed yet. The current knowledge lacks a clear consensus on the sites that are most suitable for stimulation of the auricular branch of the vagus nerve. When the studies up to today are evaluated, it can be said that the concha and inner tragus appear to be good locations for vagal neuromodulation [23]. We used a new design ear set to stimulate both parts of the auricular vagus bilaterally.

Since the underlying causes of FMS include ANS dysfunction and mostly sympathetic nervous system hyperactivity, we focused on vagus nerve stimulation, in addition to exercise treatment that would impact ANS. For this reason, the control group set up was only assigned an exercise treatment program whereas the treatment group received vagus nerve stimulation alongside the exercise treatment program. All patients were asked to complete VAS, FIQ, SF-36, Beck Depression, and Beck Anxiety Questionnaires before and after treatment in order to evaluate the efficiency of the treatment. One of the shortcomings in our study is to not evaluate the ANS activity by heart rate variability or other methods despite FMS being associated with ANS dysfunction.

There is a myriad of studies in the literature investigating the efficiency of exercise in FMS treatment; however, there is no fully completed study on the usage of vagus nerve stimulation on FMS treatment.

A systematic review by Brosseau et al. focuses on the importance of aerobic exercise in FMS treatment, stating that aerobic exercise stimulates the endogenous analgesic system and generates beneficial impacts by improving deep sleep state and life quality [24].

Wennemer et al. studied exercise training with FMS patients during an 8-week multidisciplinary treatment program. Within this study, the exercise training involves flexibility, strengthening, stretching, Tai Chi, resting, and aerobic exercise. Researchers reported that this multidisciplinary treatment program impacted physical functionality and functional capacity, which is evaluated with a 6-minute walking test [25].

In another study, conducted with 27 patients with diagnosed FMS, the effects of callisthenic exercise training, conventional strengthening exercises, and stretching exercises were compared. Patients were divided into two groups. The callisthenic exercise program was assigned to the first group, and strengthening, stretching, and posture exercises, involving neck and back muscles, were assigned to the second group. Patients were asked to complete the assigned exercises 3 days a week for an 8-week period. The number of sore points, level of physical functionality, life quality, flexibility, and grip strength were evaluated before and after the exercises. The results demonstrated that both groups displayed significant improvement [4].

Another study, conducted at Dokuz Eylül University, divided 32 patients with diagnosed FMS according to ACR criteria into two groups. The first group completed stretching, strengthening, and posture exercises, and the second group completed postisometric relaxation, remedial exercises, and active mobilization exercises 3 times a week for 3 weeks. Patients were evaluated before and after the treatment, and both groups revealed statistically significant improvement [26]. Researches about efficacy of exercise on FMS show that it should be preferred for treatment.

The control group in our study revealed similar outcomes as those in the literature. Patients were evaluated before and after treatment, and the results demonstrated that exercise had a statistically significant impact on depression, anxiety, pain, physical function, physical role difficulty, emotional role difficulty, energy/liveliness/vitality, mental health, social functionality, and general health perception.

The studies illustrated that transcutaneous vagus nerve stimulation, which is a noninvasive stimulation method through the auricular branch of the vagus nerve, generates hopeful results in the treatment of a major depressive disorder. Involving 17 patients and 21 patients in the control group, a study by Fang et al. included 4 weeks of vagus nerve stimulation. Findings before and after the treatment were evaluated with the Hamilton Depression Assessment Scale. Scores revealed significant improvement as a result of the 4-week treatment program [27].

In another study, conducted to determine the potential impact of transcutaneous stimulation of the periodic path of the vagus nerve on pain, 38 healthy volunteers were assigned tonic thermoanalgesic paradigms that show a constant pain component. Volunteers were later measured using a quantitative sense test by a trained experimenter, according to standard protocols and standard equipment. Each subject participated in two experimental sessions on different days and in randomized orders with active vagus nerve stimulation or pseudo stimulation. As a result of the active vagus nerve stimulation, it was observed that mechanic and compression pain thresholds increased and mechanic pain sensibility decreased. The study, therefore, suggests that active vagus nerve stimulation significantly decreases pain classifications, compared to the control group, and caused no other side effects on cardiac and respiratory activities [13].

In a study that evaluates the efficiency of transcutaneous stimulation of the auricular branch of the vagus nerve for chronic migraine treatment, 46 patients with chronic migraines were randomized to receive 25 Hz. or 1 Hz. stimulation of the sensory vagal region in the left ear for 4 hours a day for a 3-month period. In total, 40 out of the original 46 patients completed the study. As a result, the headache days of the patients in the 1 Hz. group experienced a significantly larger drop, in comparison with those in the 25 Hz. group. Overall, 29.4% of the patients in the 1 Hz. group experienced a reduction greater than 50% while patients in the 25 Hz. group experienced a reduction of 13.3%. Pain assessment scores also revealed significant improvement in both groups without any group distinctions. No side effects were encountered in this treatment [28].

Bauer et al. conducted a randomized double-blind controlled study that measures the efficiency of transcutaneous vagus nerve stimulation for 20 weeks in drug-resistant epilepsy patients. The purpose of the study was to illustrate the potential improvements to health as a result of 20 weeks of transcutaneous vagus nerve stimulation compared to the control group, which was under drug therapy in terms of decreasing seizure frequency. At the end of the treatment, no statistically significant drop in the average number of seizures between the groups was observed; yet, a significant decrease in the seizure frequency of patients was, in fact, found [29]. All these studies related with depression, pain, epilepsy, and migraine give clues about the central neuromodulatory effects of auricular vagus nerve stimulation.

With the exception of our study, there are currently no other completed studies investigating the use of vagus nerve stimulation for fibromyalgia. However, there is an ongoing study which is estimated for completion. In this study, 20 patients between the ages of 20 and 60, with diagnosed FMS according to 2010 ACR diagnostic criteria, are randomly divided into two groups. The control group receives pharmacological physical therapy methods, while the treatment group receives Percutaneous Electrical Neural Region Stimulation through the auditory canal. The neurostimulation system that is implemented in the ear for five days is changed once a week for four weeks. The assessment methods are implemented two weeks prior to treatment commences and two weeks after the treatment is completed. This study began in 2017 and is ongoing [30].

Additionally, in a study that investigates the efficiency of exercise in the treatment of sleep apnea, which is a disease that stems from ANS dysfunctions, 74 patients between the ages of 40 and 80 were divided into two groups and treatment group participated in an exercise program for 9 months. The control group did not do any kind of exercise. Cardiac autonomic functions of the patients were then measured using the method of heart rate variability. Patients in the exercise treatment group also revealed statistically significant improvements [31].

An examination of the literature shows that various evaluations have been carried out to investigate the efficiency of exercise treatment and vagus nerve stimulation in major depressive disorders, epilepsy, migraines, chronic pain, sleep apnea, and many other diseases that manifest through ANS dysfunctions. Since ANS dysfunction is included in the etiology of FMS and due to a lack of completed studies on the efficiency of vagus nerve stimulation for this condition, we carried out this study, aiming to illustrate the efficiency of auricular vagus nerve stimulation in FMS patients for pain and life quality. We assessed 60 patients in the study; however, only 52 were able to complete the full program. We divided patients into two groups; 27 received home-based exercise programs along with auricular vagus nerve stimulation (treatment group), while the control group was prescribed only the home-based exercise programs. The questionnaires showed that both groups yielded significant improvements in all findings at the end of 20 sessions. An examination of the relationship between groups before and after the treatment showed that the treatment group had better results; however, the SF-36 Questionnaire only revealed statistically significant differences in physical function, social functionality, and pain subparameters. We do not have sufficient data to prove the superiority of vagus nerve stimulation compared to exercise only because statistically detailed calculation of the intergroup relations is quite challenging and limited time was available. We did not encounter any side effects due to vagus nerve stimulation, and it was well tolerated by the patients.

The impact of exercise treatment on FMS is clearly demonstrated in our study, the result of which is in line with the previous studies found in the literature. Employment of vagus nerve stimulation, in addition to exercise treatment in FMS, improves the efficiency of treatment. Aside from the parameters we assessed in our study, vagus nerve stimulation may provide additional contributions to the exercise treatment itself. Vagus nerve stimulation could be an option for patients who cannot exercise. Therefore, further studies with more participants are required in order to further evaluate the impact of exercise and vagus nerve stimulation on FMS.

## Figures and Tables

**Table 1:** Descriptive statistics of identified variables for the control group (*n* = 25).

		*n* (%)			*n* (%)
Marital status	Married	21 (84.0)	Occupation	Housewife	12 (48.0)
	Single	14 (16.0)		Canteen employee	1 (4.0)
Level of education	Elementary school	7 (28.0)		Accountant	1 (4.0)
	Junior high school	4 (16.0)		Student	2 (8.0)
	High school	3 (12.0)		Teacher	6 (24.0)
	University	11 (44.0)		Professional cook	1 (4.0)
Complaints	Pain	18 (72.0)		Insurance professional	1 (4.0)
	Pain, numbness	1 (4.0)		Cleaning staff	1 (4.0)
	Pain, numbness, weakness	1 (4.0)	Accompanying diseases/conditions	Asthma	1 (4.0)
	Pain, numbness, fatigue	1 (4.0)		Herniated disc	2 (8.0)
	Pain, fatigue	4 (16.0)		Herniated disc (neck, lower back)	1 (4.0)
Daily sporting act.	None	20 (80.0)		High blood pressure	2 (8.0)
	Walking	5 (20.0)		None	19 (76.0)
Duration of pain	≤1 year	12 (48.0)			
	1-8 years	7 (28.0)			
	≥8 years	6 (24.0)			

**Table 2:** Descriptive statistics of identified variables for the treatment group (*n* = 27).

		*n* (%)			*n* (%)
Marital status	Married	18 (66.7)	Occupation	Retired	1 (3.7)
	Single	6 (22.2)		Housewife	14 (51.9)
	Other	3 (11.1)		Financial officer	1 (3.7)
Level of education	Elementary school	9 (33.3)		Nurse	4 (14.8)
	Junior high school	1 (3.7)		Mechanical engineer	1 (3.7)
	High school	9 (33.3)		Accountant	2 (7.4)
	University	7 (26.0)		Student	1 (3.7)
	Master's/PhD	1 (3.7)		Secretary	1 (3.7)
Complaints	Pain	18 (66.7)		Textile	2 (7.4)
	Pain, weakness	1 (3.7)	Accompanying diseases/conditions	Allergic asthma	1 (3.7)
	Pain, numbness	2 (7.4)		Anxiety	1 (3.7)
	Pain, numbness, fatigue	1 (3.7)		Herniated disc	3 (11.1)
	Pain, burning	2 (7.4)		Straightening of cervical lordosis	2 (7.4)
	Pain, fatigue	1 (3.7)		Goiter	1 (3.7)
	Pain, fatigue, insomnia	2 (7.4)		Hypoglycemia	1 (3.7)
Daily sporting act.	Stretching ex.	1 (3.7)		Reflux	1 (3.7)
	Pilates	2 (7.4)		High blood pressure	1 (3.7)
	Pilates, fitness	1 (3.7)		None	16 (59.3)
	Pilates, walking	1 (3.7)	Duration of pain	≤1 year	8 (29.6)
	Walking	6 (22.2)		1-8 years	9 (33.3)
	None	16 (59.3)		≥8 years	10 (37.1)

**Table 3:** Comparison of measurement values in the control group before and after the exercise.

	Before treatment Average ± SD interquartile (IQR)	After treatment Average ± SD interquartile (IQR)	*t*, *Z*	*p*
Beck Depression Scale	16.76 ± 10.63	11.92 ± 7.06	4.132^∗^	**<0.001**
Beck Anxiety Scale	20.00 (16.50)	13.00 (11.00)	3.636	**<0.001**
Fibromyalgia Impact Questionnaire	54.48 ± 18.81	41.93 ± 18.15	5.763^∗^	**<0.001**
VAS	5.67 ± 2.10	3.45 ± 1.73	7.097^∗^	**<0.001**

SF 36 Scale				
Physical Function	70.00 (17.50)	85.00 (22.50)	3.619	**<0.001**
Physical Role Difficulty	25.00 (50.00)	50.00 (50.00)	3.225	**0.001**
Emotional Role Difficulty	33.33 (66.67)	66.67 (33.33)	2.336	**0.019**
Energy/Liveliness/Vitality	40.00 (37.50)	50.00 (20.00)	3.791	**<0.001**
Mental Health	50.24 ± 19.26	62.40 ± 16.93	3.919^∗^	**0.001**
Social Functionality	62.50 (25.00)	75.00 (18.75)	3.824	**<0.001**
General Health Perception	42.60 ± 21.51	52.20 ± 15.46	4.129	**<0.001**
Pain	41.10 ± 21.06	59.50 ± 15.28	5.451^∗^	**<0.001**

^∗^ *t*-test results are provided.

**Table 4:** Comparison of measurement values in the treatment group before and after the treatment.

	Before treatment Average ± SD interquartile (IQR)	After treatment Average ± SD interquartile (IQR)	*t*, *Z*	*p*
Beck Depression Scale	16.00 (12.00)	8.00 (12.00)	3.660	**<0.001**
Beck Anxiety Scale	18.00 (13.00)	13.00 (13.00)	3.692	**<0.001**
Fibromyalgia Impact Questionnaire	61.98 ± 18.45	37.27 ± 19.48	5.883^∗^	**<0.001**
VAS	6.17 ± 2.58	2.56 ± 1.91	5.859^∗^	**<0.001**

SF 36 Scale				
Physical Function	65.00 (25.00)	80.00 (25.00)	4.024	**<0.001**
Physical Role Difficulty	0.00 (50.00)	75.00 (75.00)	3.116	**0.002**
Emotional Role Difficulty	0.00 (66.67)	100.00 (66.67)	2.764	**0.006**
Energy/Liveliness/Vitality	28.70 ± 21.64	57.59 ± 22.97	5.153^∗^	**<0.001**
Mental Health	46.37 ± 19.09	65.33 ± 20.66	4.265^∗^	**0.001**
Social Functionality	47.22 ± 26.02	69.91 ± 22.80	3.583^∗^	**0.001**
General Health Perception	33.89 ± 19.38	56.85 ± 21.45	6.126	**<0.001**
Pain	27.87 ± 21.54	58.05 ± 18.80	6.741^∗^	**<0.001**

^∗^ *t*-test results are provided.

**Table 5:** Comparison of control group and treatment group before intervention.

	Control Average ± SD interquartile (IQR)	Treatment Average ± SD interquartile (IQR)	*t*, *Z*	*p*
Beck Depression Scale	16.76 ± 10.63	17.63 ± 8.43	0.328	0.744
Beck Anxiety Scale	20.00 (16.50)	18.00 (13.00)	0.284	0.776
Fibromyalgia Impact Questionnaire	54.48 ± 18.81	61.98 ± 18.45	1.450	0.153
VAS	5.67 ± 2.10	6.17 ± 2.58	0.756	0.453

SF 36 Scale				
Physical Function	70.00 (17.50)	65.00 (25.00)	2.281	**0.023**
Physical Role Difficulty	25.00 (50.00)	0.00 (50.00)	0.908	0.364
Emotional Role Difficulty	33.33 (66.67)	0.00 (66.67)	0.520	0.603
Energy/Liveliness/Vitality	40.00 (37.50)	25.00 (35.00)	1.444	0.149
Mental Health	50.24 ± 19.26	46.37 ± 19.09	0.727	0.471
Social Functionality	62.00 ± 18.21	47.22 ± 26.02	2.386	**0.021**
General Health Perception	42.60 ± 21.51	33.89 ± 19.38	1.536	0.131
Pain	45.00 (35.00)	22.50 (32.50)	2.363	**0.018**

^∗^ *t*-test results are provided.

**Table 6:** Comparison of control group and treatment group after intervention.

	Control Average ± SD interquartile (IQR)	Treatment Average ± SD interquartile (IQR)	*t*, *Z*	*p*
Beck Depression Scale	13.00 (12.00)	8.00 (12.00)	1.357	0.175
Beck Anxiety Scale	13.00 (11.00)	13.00 (13.00)	0.513	0.608
Fibromyalgia Impact Questionnaire	41.93 ± 18.15	37.27 ± 19.48	0.890^∗^	0.378
VAS	3.45 ± 1.73	2.56 ± 1.91	1.766^∗^	0.084

SF 36 Scale				
Physical Function	85.00 (22.50)	80.00 (25.00)	1.383	0.167
Physical Role Difficulty	50.00 (50.00)	75.00 (75.00)	0.680	0.496
Emotional Role Difficulty	66.67 (33.33)	100.00 (66.67)	1.299	0.194
Energy/Liveliness/Vitality	51.60 ± 19.46	57.59 ± 22.97	1.011^∗^	0.317
Mental Health	62.40 ± 16.93	65.33 ± 20.66	0.557^∗^	0.580
Social Functionality	75.00 (18.75)	75.00 (37.50)	0.580	0.562
General Health Perception	52.20 ± 15.46	56.85 ± 21.45	0.508^∗^	0.614
Pain	59.50 ± 15.28	58.06 ± 18.80	0.303^∗^	0.763

^∗^ *t*-test results are provided.

## Data Availability

The [DATA TYPE] data used to support the findings of this study are available from the corresponding author upon request.

## References

[1] Ozcan D. S., Oken O., Aras M., Koseoglu B. F. (2014). Vitamin D levels in women with fibromyalgia and relationship between pain, depression and sleep. *Türkiye Fiziksel Tıp ve Rehabilitasyon Dergisi*.

[2] Aslan Ş., Başaran S., Çeliker R. (2012). *Fiziksel Tıp ve Rehabilitasyonda Yeni Ufuklar Fibromiyalji*.

[3] Gürer G., Şendur Ö. F. (2006). *Corelations of Clinical Features and Findings in Fibromyalgia Patients, Adnan Menderes Üniversitesi Tıp Fakültesi Fiziksel Tıp Ve*.

[4] Yürük Ö., Gültekin Z. (2008). Fibromiyalji sendromu olan kadınlarda iki farklı egzersiz programının karşılaştırılması. *Fizyoterapi Rehabilitasyon*.

[5] Doğan Ş., Ay S., Evcik D. (2011). Current approaches in the treatment of fibromyalgia. *Yeni Tıp Dergisi*.

[6] Gür A. (2008). Etiopathogenesis in fibromyalgia. *Türkiye Fiziksel Tıp ve Rehabilitasyon Dergisi, Türkiye Fiziksel Tıp ve Rehabilitasyon Dergisi*.

[7] Toprak Celenay S., Anaforoglu Kulunkoglu B., Yasa M. E. (2017). A comparison of the effects of exercises plus connective tissue massage to exercises alone in women with fibromyalgia syndrome: a randomized controlled trial. *Rheumatology International*.

[8] Kingsley J. D. (2012). Autonomic dysfunction in women with fibromyalgia. *Arthritis Research and Therapy*.

[9] Martínez-Martínez L.-A., Mora T., Vargas A., Fuentes-Iniestra M., Martínez-Lavín M. (2014). Sympathetic nervous system dysfunction in fibromyalgia chronic fatigue syndrome, irritable bowel syndrome and interstitial cystitis. *Journal of Clinical Rheumatology*.

[10] Kulshreshtha P., Gupta R., Yadav R. K., Bijlani R. L., Deepak K. K. (2012). A comprehensive study of autonomic dysfunction in the fibromyalgia patients. *Clinical Autonomic Research*.

[11] Yuan H., Silberstein S. D. (2016). Vagus nerve and vagus nerve stimulation, a comprehensive review: part I. *Headache*.

[12] Yuan H., Silberstein S. D. (2016). Vagus nerve and vagus nerve stimulation, a comprehensive review: part II. *Headache*.

[13] Busch V., Zeman F., Heckel A., Menne F., Ellrich J., Eichhammer P. (2013). The effect of transcutaneous vagus nerve stimulation on pain perception - An experimental study. *Brain Stimulation*.

[14] Sarmer S., Ergin S., Yavuzer G. (2000). The validity and reliability of the turkish version of the fibromyalgia impact questionnaire. *Rheumatology International*.

[15] Price D. D., McGrath P. A., Rafii A., Buckingham B. (1983). The validation of visual analogue scales as ratio scale measures for chronic and experimental pain. *Pain*.

[16] Demiral Y., Ergor G., Unal B. (2006). Normative data and discriminative properties of short form 36 (SF-36) in turkish urban population. *BMC Public Health*.

[17] Koçyiğit H., Aydemir Ö., Fişek G., Ölmez N., Memiş A. (1999). Kısa form-36’nın Türkçe versiyonunun güvenilirliği ve geçerliliği. *İlaç ve Tedavi Dergisi*.

[18] Hisli N. (1988). A validation study of Beck depression inventory [in Turkish]. *Turkish Journal of Psychology*.

[19] Ulusoy M., Şahin N., Erkmen H. (1998). Turkish version of the beck anxiety inventory: psychometric properties. *Journal of Cognitive Psychotherapy*.

[20] Özkan N. (2017). Complementary approach in fibromyalgia syndrome. *Journal of Complementary Medicine, Regulation and Neural Therapy*.

[21] Sindel D., Saral İ., Esmaeilzadeh S. (2012). Management approaches in fibromyalgia syndrome. *Türkiye Fiziksel Tıp ve Rehabilitasyon Dergisi*.

[22] Buturak V., Bakar B. (2014). İlaç tedavisine dirençli depresyonda bir alternatif tedavi yöntemi: vagal sinir uyarımı. *Journal of Mood Disorders*.

[23] Butt M. F., Albusoda A., Farmer A. D., Aziz Q. (2019). The anatomical basis for transcutaneous auricular vagus nerve stimulation. *Journal of Anatomy*.

[24] Brosseau L., Wells G. A., Tugwell P. (2008). Ottawa panel evidence-based clinical practice guidelines for aerobic fitness exercises in the management of fibromyalgia: part 1. *Physical Therapy*.

[25] Wennemer H. K., Borg-Stein J., Gomba L. (2006). Functionally oriented rehabilitation program for patients with fibromyalgia. *American Journal of Physical Medicine & Rehabilitation.*.

[26] Genç A., Sağıroğlu E. (2002). Comparison of two different exercise programs in fibromiyalgia treatment. *Turkish Journal of Physical Medicine and Rehabilitation*.

[27] Fang J., Egorova N., Rong P. (2017). Early cortical biomarkers of longitudinal transcutaneous vagus nerve stimulation treatment success in depression. *NeuroImage: Clinical*.

[28] Straube A., Ellrich J., Eren O., Blum B., Ruscheweyh R. (2015). Treatment of chronic migraine with transcutaneous stimulation of the auricular branch of the vagal nerve (auricular t-VNS): a randomized, monocentric clinical trial. *The Journal of Headache and Pain*.

[29] Hamer H. M., Bauer S. (2019). Lessons learned from transcutaneous vagus nerve stimulation (tVNS). *Epilepsy Research*.

[30] Gebre M., Woodbury A., Napadow V. (2018). Functional magnetic resonance imaging evaluation of auricular percutaneous electrical neural field stimulation for fibromyalgia: protocol for a feasibility study. *JMIR Research Protocols*.

[31] Berger M., Raffin J., Pichot V. (2019). Effect of exercise training on heart rate variability in patients with obstructive sleep apnea: a randomized controlled trial. *Scandinavian Journal of Medicine & Science in Sports.*.

